# mir-329 restricts tumor growth by targeting grb2 in pancreatic cancer

**DOI:** 10.18632/oncotarget.7375

**Published:** 2016-02-14

**Authors:** Xinjing Wang, Xiongxiong Lu, Tian Zhang, Chenlei Wen, Minmin Shi, Xiaomei Tang, Hao Chen, Chenghong Peng, Hongwei Li, Yuan Fang, Xiaxing Deng, Baiyong Shen

**Affiliations:** ^1^ Research Institute of Pancreatic Disease, Ruijin Hospital, School of Medicine, Shanghai Jiao Tong University, Shanghai, China; ^2^ Pancreatic Disease Centre, Ruijin Hospital, School of Medicine, Shanghai Jiao Tong University, Shanghai, China; ^3^ Shanghai Institute of Digestive Surgery, Ruijin Hospital, School of Medicine, Shanghai Jiao Tong University, Shanghai, China

**Keywords:** pancreatic cancer, miR-329, GRB2, apoptosis

## Abstract

Pancreatic cancer is one of the most lethal malignancies worldwide. To illustrate the pathogenic mechanism(s), we looked into the expression and function of miR-329 associated with pancreatic cancer development. It was found that miR-329 expression was downregulated in the pancreatic cancer patients who demonstrated significantly shorter overall survival than the patients having upregulated expression. Also, more advanced pT stage cases were observed in the low miR-329 expression group of patients. Interestingly, our studies uncovered that miR-329 overexpression inhibited proliferation and induced apoptosis of pancreatic cancer cells, in contrast the miR-329 inhibitor reversed this phenomenon dramatically. Additionally, overexpression of miR-329 significantly limited tumor growth in the xenograft model. In the mechanistic study, we identified GRB2 as a direct target of miR-329 in pancreatic cancer cells, and expression of GRB2 was inversely correlated with miR-329 expression in pancreatic cancer patients. Furthermore, GRB2 overexpression in cell line and xenograft model dramatically diminished miR-329 mediated anti-proliferation and apoptosis induction, indicating that GRB2/pERK pathway was mainly downregulated by miR-329 expression. In general, our study has shed light on miR-329 regulated mechanism and, miR-329/GRB2/pERK is potential to be targeted for pancreatic cancer management.

## INTRODUCTION

Pancreatic cancer is the malignancy showing extremely poor prognosis [1-4]. Despite increasing surgical and clinical treatments, the median survival rate under 1 year of resected patients is around 18% and the 5-year overall survival remains about 6% [4-6]. Therefore, it is necessary to deeply understand the molecular basis and biology of pancreatic cancer for providing novel diagnostic manner and therapeutic strategy.

MicroRNAs (miRNAs) are endogenous small noncoding single-stranded RNAs which negatively regulate gene expression through mRNA degradation or translation repression [7-9] It has been known that miRNAs function as crucial regulators in many biological processes involved in carcinogenesis, such as proliferation, differentiation, apoptosis, and metabolism [10-12]. It has been shown that miRNAs can either function as oncogene (i.e. miR-155) or tumor suppressors (i.e. miR-497 and miR-29c) in pancreatic cancer development [13-16]. In aim of developing effective diagnosis or therapies for pancreatic cancer, it is interesting to understand the function and mechanism of newly discovered miRNAs that regulate pancreatic cancer development. By a comprehensive study of the published literatures [17-21], we have uncovered that miR-329, which is located on 14q32.31 [17], is potential to regulate cancer progression. It was found that miR-329 downregulated E2F1 expression and inhibited glioma cells proliferation [18], also, miR-329 suppressed tumor invasion and growth by targeting TIAM1 in gastric cancer [19]. Recently, miR-329 was found to inhibit cell growth and motility in neuroblastoma by targeting LSD1 [20, 21].

To illustrate the expression and function of miR-329 in pancreatic cancer, we have investigated the miR-329 mediated regulation in this severe malignancy. Firstly, we evaluated the expression of miR-329 in pancreatic cancer patients. It was demonstrated that miR-329 was downregulated in the patients who showed significantly shorter overall survival than the patients having upregulated miR-329 expression. In particular, more advanced pT stage cases were found in the low miR-329 expression group of patients when compared to the high miR-329 expression group of patients. Subsequently, our functional study showed that overexpression of miR-329 significantly attenuated cell proliferation in both pancreatic cancer cells and xenograft model. Lastly, we utilized bioinformatic analysis to identify growth factor receptor-bound protein 2 (GRB2) was a direct target of miR-329. The GRB2/pERK pathway was mainly involved in the miR-329 mediated anti-proliferation in pancreatic cancer.

## RESULTS

### MiR-329 was downregulated in the pancreatic cancer patients showing poor prognosis

**Figure 1:**
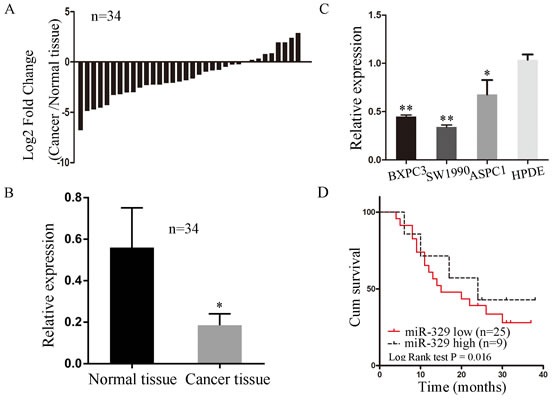
MiR-329 was downregulated in the pancreatic cancer patients showing poor survival **A.** MiR-329 expression was investigated in the cancer tissue compared with its adjacent normal tissues of 34 patients by qPCR. Results are presented as log 2 fold change of cancer tissues relative to normal tissues. **B.** The average of miR-329 expression level in cancer and normal tissue of 34 patients were shown by bar graph. Data are shown 2^−ΔΔCt^ values. **C.** MiR-329 expressions of pancreatic cancer cell lines SW1990, BXPC3 and ASPC1 compared with normal pancreatic ductal cell line HPDE were grafted by bar plots. **D.** The survival of pancreatic cancer patients were analyzed by Kaplan-Meier and log-rank tests. Patients were characterized by high and low expression of miR-329 based on the qPCR results shown in the panel A (high expression > 0; low expression < 0). Standard deviation of the mean was plotted for bar charts. (* *P* < 0.05, ** *P* < 0.01).

To uncover the expression level of miR-329 in pancreatic cancer, the RNA level was evaluated by real-time PCR in the cancer tissue of 34 patients. We found the expression level of miR-329 in the cancer tissue was significantly lower when compared to the paired normal tissue ( *P* < 0.05) (Figure 1A and 1B). In addition, we analyzed the expression of miR-329 in the pancreatic cancer cell lines BXPC3, SW1990 and ASPC1. It was shown that miR-329 expression was highly decreased than the normal pancreatic ductal cell HPDE (Figure 1C).

In our further study, we investigated the correlation between miR-329 expression and clinicopathologic characteristics of pancreatic cancer patients. It was shown in the Table 1 that the expression of miRNA-329 was not related to gender ( *P* = 0.48), age ( *P* = 0.12), pN stage (*P* = 0.36), perineural invasion (*P* = 0.39), or pTNM stage ( *P* = 0.08). However, miR-329 expression was significantly associated with pT stage (*P* = 0.02). Furthermore, we found that the patients showing high level of miR-329 expression had significantly higher survival rate than the patients showing low miR-329 expression (*P* < 0.05; Figure 1D). In addition, the miR-329 expression was treated as a continuous variable to generate the *p* value of the correlation between miR-329 expression and survival was 0.014. In multivariate models including pT stage, the *p* value for miR-329 expression level was 0.005. These results indicate that miR-329 expression is strongly associated with the prognosis of pancreatic cancer patients.

**Table 1:** Correlation between miR-329 expression and clinicopathologic characteristics of 34 pancreatic cancer patients

Clinicopathologic characteristics	Low expression^a^ (*n* = 25)	High expression^b^ (*n* = 9)	*P* value
Gender			0.48
Male	16	5	
Female	9	4	
Age			0.12
< 60	12	7	
≥ 60	13	2	
pT stage			0.04
T1	7	7	
T2	12	2	
T3	6	0	
pN stage			0.70
N0	12	3	
N1	13	6	
pTNM stage			0.30
IA	3	3	
IB	5	3	
IIA	4	0	
IIB	13	3	
Perineural invasion			0.39
No	6	1	
Yes	19	8	

a,b According to the results shown in the Figure 1A, we defined the high expression group of patients as the ratio of the cancer/normal miR-329 expression level greater than 1 and the low expression group as less than 1.

### MiR-329 inhibited proliferation of pancreatic cancer cells

**Figure 2:**
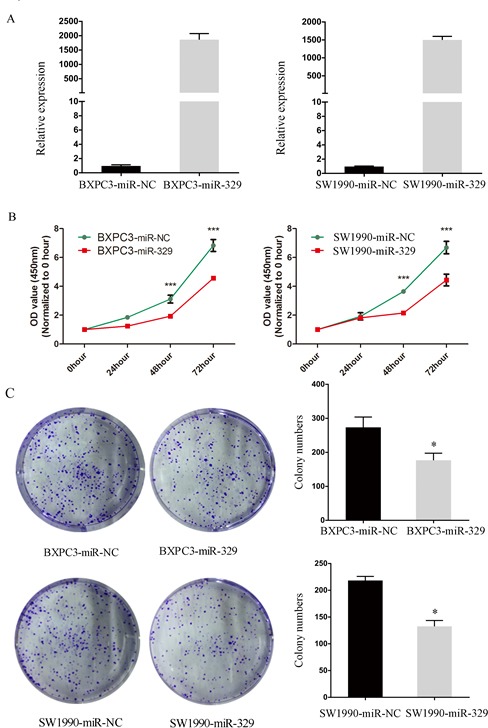
MiR-329 inhibited proliferation of pancreatic cancer cells **A.** MiR-329 expressions in cell lines SW1990 and BXPC3 were quantified by qPCR. **B.** Cell proliferation assay in SW1990 and BXPC3 transfected with miR-329 mimics were shown. **C.** Colony formation assay and statistical analysis of SW1990 and BXPC3 transfected with miR-329 mimics were exhibited. Standard deviation of the mean was plotted for bar charts. (* *P* < 0.05, *** *P* < 0.001).

To explore the potential role of miR-329 in pancreatic cancer, we overexpressed miR-329 in the pancreatic cancer cell lines by transfecting miR-329 mimics or miR-329 scramble (miR-NC) into BxPC3 and SW1990. The upregulated level of miR-329 was confirmed by qPCR after transfection (Figure 2A). Then we utilized CCK8 analysis and colony formation assay to evaluate the effect of miR-329 expression on cell proliferation. As indicated in Figure 2B and 2C, miR-329 overexpression significantly inhibited cell growth and colony formation of both BXPC3 and SW1990 cells. Additionally, we observed the growth and colony formation of pancreatic cancer primary cells overexpressing miR-329 were dramatically inhibited (Supplementary Figure 1A-1C). On the other hand, the treatment of miR-329 inhibitor on ASPC1 significantly enhanced cell growth (Supplementary Figure 2A, 2B). Taken these results together, miR-329 is capable of suppressing proliferation of the pancreatic cancer cells *in vitro*.

### MiR-329 restricted tumor growth in xenograft mouse model

**Figure 3:**
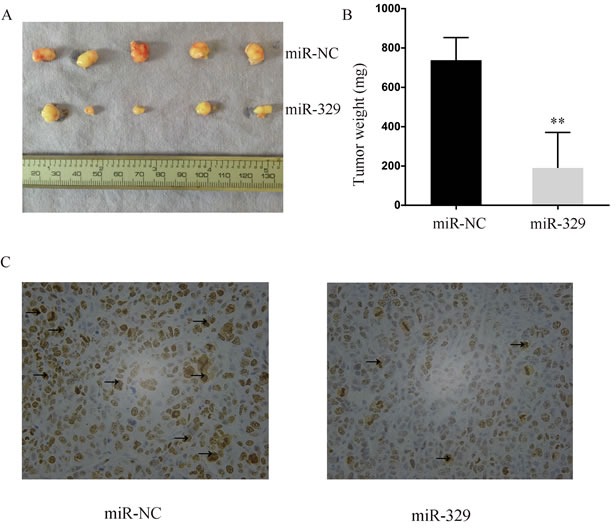
MiR-329 attenuated tumor growth *in vivo* **A.** The size of the xenograft tumor harvested from nude mice was evaluated. **B.** The weight of the xenograft tumor was shown by bar graph. **C.** Representative photographs of immunohistochemical analysis of Ki-67 expression in the xenograft tumor collected from nude mice. The positive staining was delineated by the arrow. Standard deviation of the mean was plotted for bar charts. (** *P* < 0.01)

Given that miR-329 inhibited cell proliferation *in vitro*, we next evaluated the suppressive role of miR-329 in tumor growth by utilizing xenograft mouse model. The SW1990-miR-329 and SW1990-miR-NC cells were injected into nude mice subcutaneously, then the growth of xenograft was monitored. It was shown that the tumor volume and weight in the miR-329 overespression group were remarkably reduced than the control group (Figure 3A and 3B). Furthermore, we examined the proliferative index of Ki-67 in the tumor graft by IHC which demonstrated that Ki-67 positive rate was significantly lower (*p* < 0.01) in the SW1990-miR-329 tumor (45.6%±6.5%) compared with the SW1990-miR-NC tumor (75.6%±5.5%) shown in the Figure 3C. Thus, miR-329 is capable of restricting tumor growth *in vivo*.

### MiR-329 induced pancreatic cancer cell apoptosis

**Figure 4:**
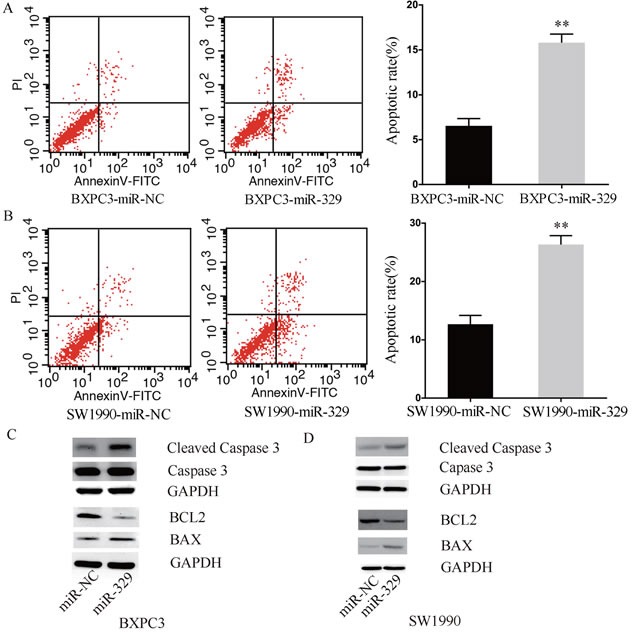
MiR-329 induced pancreatic cancer cell apoptosis **A.-B.** Flow cytometry and apoptotic rate analyses of the cell line BXPC3 and SW1990 were shown, respectively. **C.-D.** Expressions of apoptosis related proteins i.e. cleaved Caspase-3, BAX and BCL2 of BXPC3 and SW1990 were examined by western blot assay, respectively. GAPDH was used as a reference. Standard deviation of the mean was plotted for bar charts. (** *P* < 0.01).

In the further study, we investigated whether miR-329 mediated inhibition of cell proliferation was due to induction of apoptosis. As shown in Figure 4A and 4B respectively, by flow cytometry analysis, the population of apoptotic cells was significantly increased in the BXPC3 and SW1990 cells transfected with miR-329 mimics when compared to the control (miR-NC) group. Moreover, the similar phenomenon was observed in the primary cells transfected with miR-329 mimics (Supplementary Figure 1D). However, downregulation of miR-329 by its inhibitor significantly limited cell apoptosis of ASPC1 (Supplementary Figure 2C).

Based on the results that miR-329 induced cell apoptosis, we next performed western blot to evaluate the expression level of apoptosis related proteins such as cleaved Caspase-3, BAX and BCL2 in the pancreatic cancer cells overexpressing miR-329. Interestingly, in both BxPC3 and sw1990 cells, the levels of apoptosis prone protein BAX and cleaved Caspase-3 were highly enhanced in the miR-329 overexpression group, whereas the level of anti-apoptotic protein BCL2 was dramatically decreased (Figure 4C and 4D). Taken together, miR-329 is able to induce pancreatic cancer cell apoptosis, which in hence lead to cell growth inhibition.

### MiR-329 directly targets the 3′UTR of GRB2 and pERK pathway

**Figure 5:**
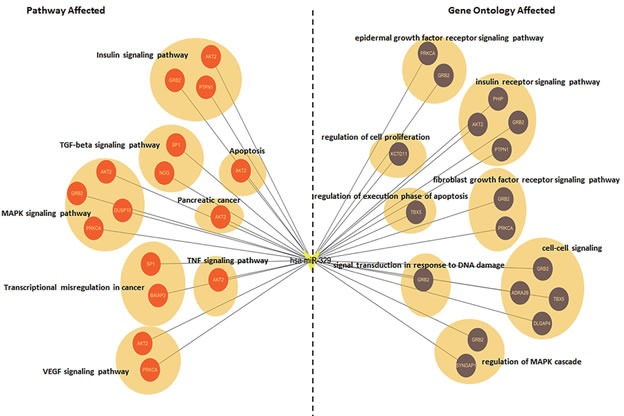
Target prediction and analysis of miR-329 At left of the network, the genes annotated by the pathway analysis were shown. At right of the network, the genes analyzed by the ontology were exhibited. The circle represents target gene.

To elucidate the mechanism(s) that are involved in miR-329 mediated cell apoptosis, we explored the potential targets of miR-329 by the bioinformatic tool TargetScan (http://www.targetscan.org/; Supplementary Table 1). Additionally, gene ontology (GO) analysis and KEGG database were utilized to illustrate the biological function associated with the significant pathways for the potential target genes of miR-329 (Supplementary Table 2 and Table 3). Based on the bioinformatic analysis, we generated the miR-329-target network including the targets that play vital roles in cell proliferation and apoptosis (Figure 5).

**Figure 6:**
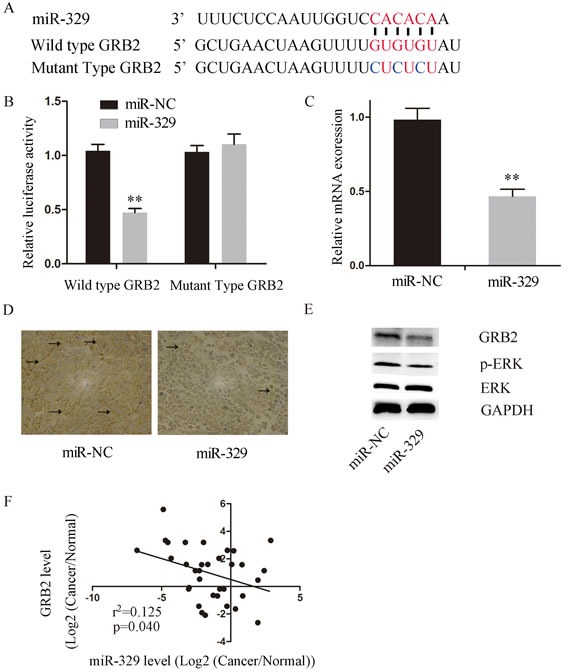
MiR-329 directly targeted the 3′UTR of GRB2 and inhibited GRB2/ pERK pathway **A.** The upper levels shows the sequences of wide type GRB2 3′UTR and the binding site of miR-329, the lower level shows the sequence of GRB2 3′UTR binding site of the created GRB2 mutant. **B.** The luciferase activity of wild type GRB2 were significantly decreased by miR-329 expression when compared to the mutant. **C.** The mRNA expression of GRB2 in the SW1990 cells overexpressing miR-329 by qPCR. **D.** Representative photographs of immunohistochemistry analysis of GRB2 expression in the xenograft tumor collected from nude mice. The positive staining was delineated by the arrow. **E.** GRB2 and pERK expressions were examined by western blot assay in the SW1990 cells transfected with miR-329 mimics and control. GAPDH was used as a reference. **F.** Overexpression of GRB2 partially inhibited miR-329 induced anti-proliferation. The SW1990 cells were simultaneously transfected with miR-329 mimics and GRB2 plasmid and then monitored by the proliferation assay. **G.** The inverse correlation between GRB2 and miR-329 expressions of 34 pancreatic cancer patients was demonstrated. Standard deviation of the mean was plotted for bar charts. (** *P* < 0.01).

By screening the targets, we uncovered GRB2 is a potential direct target of miR-329 based on its oncogenic role in inhibition of cell proliferation through pERK pathway. Firstly, we examined whether miR-329 directly bound to GRB2 by performing luciferase reporter assay. The wild and mutated 3′UTR of GRB2 (Figure 6A) were constructed and transfected into SW1990 cells along with miR-329 mimics or miR-NC, respectively. It was shown that the luciferase activity was dramatically inhibited by wild type GRB2 when compared to the GRB2 mutant (Figure 6B). Moreover, miR-329 overespression led to significant reduction in expression of wild type GRB2 at both mRNA and protein level (Figure 6C). Additionally, the GRB2 expression was highly reduced in the xenograft tumor tissue which exhibited high miR-329 expression (Figure 6D). Interestingly, the phosphorylated level of ERK was declined simultaneously with the reduced expression of GRB2 when miR-329 was overexpressed in pancreatic cancer cells (Figure 6E). These results suggest that miR-329 is capable of targeting GRB2/pERK pathway.

**Figure 7:**
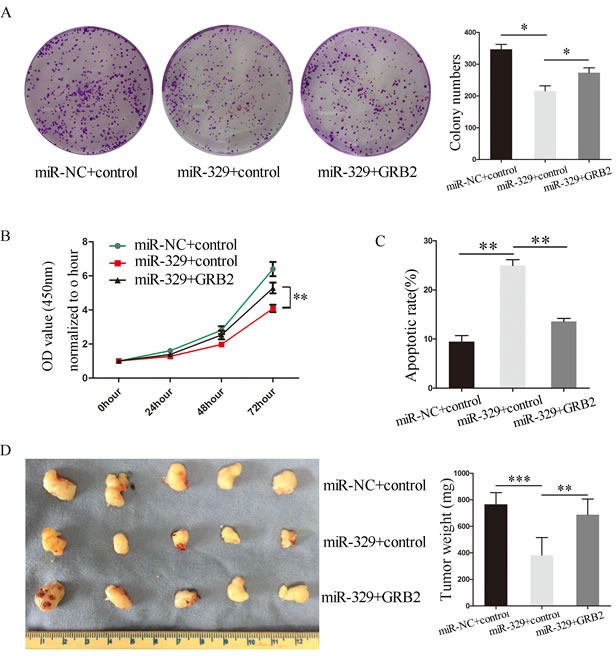
GRB2 rescued miR-329′s inhibition *in vitro* and *in vivo* Exogenous GRB2 expression in SW1990-miR-329 restored cell colony formation **A.** and proliferation **B.** which were inhibited by miR-329. **C.** Exogenous GRB2 expression in the SW1990-miR-329 cells decreased apoptotic response induced by miR-329. **D.** Exogenous expression of GRB2 restored tumor growth attenuated by miR-329 expression. Standard deviation of the mean was plotted for bar charts. (* *P* < 0.05, ** *P* < 0.01, *** *P* < 0.001).

We next investigated the relationship between miR-329 and GRB2 in pancreatic cancer patients by analyzing the expression levels of GRB2 and miR-329 in the tumor tissue collected from 34 patients. It was shown that the expression levels of miR-329 and GRB2 exhibited statistically inverse correlation by the analysis (Figure 6F). Finally, we investigated whether GRB2/pERK pathway is critically involved in miR-329 mediated inhibition by overespressing miR-329 and GRB2 in SW1990 simultaneously. It was demonstrated that exogenous GRB2 expression significantly enhanced cell proliferation and colony formation of SW1990(miR-329 + GRB2) cells in contrast to the control group (miR-329 + Control) (Figure 7A, 7B), although GRB2 expression was unable to completely inhibit miR-329 induced anti-proliferation. In addition, miR-329 mediated apoptotic response was significantly inhibited by the exogenous GRB2 expression (Figure 7C) which also resulted in dramatic inhibition of tumor growth in the xenograft model (Figure 7D). These results suggest that GRB2 is a major molecule targeted by miR-329, but additional mechanism(s) is also involved.

## DISCUSSION

MiR-329 was reported to promote tumor growth or metastasis in glioma, neuroblastoma and gastric cancer at decreased expression level [18-20]. However, the expression and function of miR-329 in pancreatic cancer is still unknown. In the present study, we found that the miR-329 expression level was downregulated in the cancer patients and was inversely correlated with disease progression. Then we uncovered that overexpression of miR-329 significantly attenuated cell proliferation in both pancreatic cancer cell lines and xenograft model. Furthermore, our study displayed that miR-329 restricted cell growth by enhancing the level of several apoptosis prone proteins. Recently, it has been reported that miR-329 played tumor suppressive role by targeting different molecules in several cancers [18-20]. Due to miR-329 mainly inhibited cell growth in pancreatic cancer, in this study, we uncovered the miR-329-target network which was closely associated with cell proliferation and apoptosis by bioinformatic analysis. Subsequently, in this network, we focused on the molecules involved in the canonic MAPK and EGFR signaling pathways that play major roles in cell growth. With this screening strategy, we discovered GRB2 which is a key signaling adaptor molecule involved in the EGFR pathway [23-25].

GRB2 contains an SH2 domain which is flanked by two SH3 domains [26, 27]. The SH2 domain recognizes the phosphotyrosine residues of activated EGFR, and the two SH3 domains bind to proline-rich sequences. Therefore, GRB2 is able to link EGFR and downstream signaling molecule (i.e. pERK) to attenuate cell growth [24, 25]. It has been illustrated that suppression of GRB2 is able to induce apoptosis in hepatic cancer [28]. Additionally, inhibition of GRB2/pERK pathway limited tumor development by promoting cell apoptosis [29, 30]. Based on the phenomena that miR-329 limited cell growth and induced apoptosis in our study, we hypothesized that miR-329 attenuated pancreatic cancer proliferation by targeting GRB2. To test this hypothesis, we created GRB2 mutant which was not downregulated by the overexpression of miR-329 when compared to the wide type GRB2. Also, we found that pERK, the downstream signaling molecule of GRB2, was decreased by miR-329 expression. These results indicate that GRB2/pERK pathway was highly involved in the miR-329 mediated cell growth inhibition. Furthermore, simultaneous overexpression of miR-329 and GRB2 in the pancreatic cancer cell and xenograft model demonstrated that the miR-329 mediated anti-proliferation was dramatically diminished by GRB2. These results suggest that GRB2/pERK pathway is mainly inhibited by miR-329, though additional anti-proliferative mechanism(s) is also involved.

In summary, our work has demonstrated that miR-329 was downregulated in pancreatic cancer and involved in cell growth by targeting GRB2. Of note, GRB2/pERK pathway is the major pathway targeted by miR-329. It is still unknown whether miR-329 is also able to regulate cancer cell migration and invasion. Further studies are needed to illustrate the complete functions of this microRNA involved in cancer development. Nevertheless, the miR-329 and its targeted molecules are potential to be the prognostic makers or targeted by the novel therapies for pancreatic cancer management.

## MATERIALS AND METHODS

### Tissue specimens and cell culture

The clinical research protocol was approved by the Ethical Committee of Ruijin Hospital, Shanghai Jiao Tong University School of Medicine. Tissue specimens were collected from 34 patients diagnosed with pancreatic cancer who had undergone pancreatic resections. Tissues were harvested freshly after samples' dissection, snap-frozen in liquid nitrogen, and finally preserved at −80.

The immortalized normal human pancreatic cell line HPDE, pancreatic cancer cell lines SW1990, BXPC3 and ASPC1 were purchased from the Type Culture Collection of the Chinese Academy of Sciences (Shanghai, China). Cells were maintained in DMEM supplemented with 10% FBS (GIBCO) at 37°C under 5% CO_2_ in a humidified chamber. Primary pancreatic cancer cells were cultured as Helene et al [32] described and maintained in RPMI 1640 with 10% FBS.

### Quantitative real-time PCR (qPCR)

Total RNA was extracted from tissues or cell lines with Trizol reagent (Invitrogen) according to the manufacture's protocol. cDNA was synthesized with 1ug RNA using reverse transcription kit (TOYOBO). SYBR Green reagent (Applied Biosystems) was used for qRT-PCR to analysis mRNA expression. U6 small nuclear RNA was used as an endogenous control for miR-329 normalization. Relative expression ratios of miR-329 or GRB2 in each paired tumor cancer to normal tissue sample were calculated using the 2^−ΔΔCt^ method. MiR-329 and U6 primers were purchased from Shanghai GenePharma Co., Ltd. The PCR primers designed for GRB2: 5′-ATTCCTGCGGGACATAGAACA-3′ (forward) and 5′-GGTGACATAATTGCGGGGAAAC-3′ (reverse); for GAPDH: 5′-GGACCTGACCTGCCGTCTAG-3′ (forward) and 5′-GTAGCCCAGGATGCCCTTGA-3′ (reverse).

### miRNA transfection

The miR-329 mimics or miR-329 inhibitors and their controls used were synthesized by Guangzhou RiboBio Co., Ltd. A day before transfection, cells were plated at a density of 1.5*10^5^ cells/well onto 6-well plates. After culturing for 24h when the cell confluence was about 70%, transfection was conducted with Lipofectamine 2000 (Invitrogen) following the manufacturer's instructions. Plasmids LV3-pGLV-H1-GFP+Puro with miR-329 mimics or control oligonucleotides were purchased from Shanghai GenePharma Co., Ltd and stable expression cell lines were established according to the manufacturer's protocol. Transfection efficiencies were evaluated by qPCR.

### Establishment of stable transfectants

Lentivirus of GRB2 in pBobi was purchased from Asia-vectorbio (Shanghai, China). Lentivirus transfection was performed according to the manufacturer's instruction to establish the stable GRB2-expressing SW1990 cells. The control was constructed similarly without inserted cDNA.

### Western blot analysis and Immunohistochemistry

Western blot was done by the standard protocol. Total protein was extracted with RIPA buffer (Solarbio, Beijing, China) in the presence of protease inhibitor cocktail (Roche Applied Science, Basel, Switzerland). The primary antibodies used were anti-GRB2 (Proteintech, Wuhan, China), anti-GAPDH (Abcam, USA), anti-ERK and anti-pERK (Cell Signaling Technology, USA). Goat anti-mouse or goat anti-rabbit IgG horseradish peroxidase (Cell Signaling Technology, USA) was used as secondary antibodies. Immunohistochemistry was performed as described previously [33]. The primary antibodies used were anti-GRB2 (Proteintech, Wuhan, China) and anti-Ki-67 (Cell Signaling Technology, USA).

### Cell proliferation assay

Proliferation assays were performed using cell counting kit 8 (CCK8, Dojindo, Japan). Cells were seeded in 96-well plates at approximately 1000 cells per well and cultured in the appropriate medium. Numbers of viable cells were quantified at each 24h interval by measuring OD450 with microplate reader (Epoch; BioTek, Winooski, VT).

### Colony formation assay

Cells were seeded into six-well plates with 1000 cells/well in 2ml culture medium and incubated for 10 days. The number of colonies was counted using a phase-contrast microscope at a magnification of 4x after staining with 0.1% crystal violet solution. We counted the colonies which containing at least 50 cells. The experiments were independently triplicated.

### Flow cytometry assay

Cells were transfected with miR-329 mimics or miR-329 inhibitors and their controls, then harvested 48h post-transfection and finally we performed the flow cytometry with the AnnexinV/PI double staining kit (BD Biosciences, USA) according to the manufacturer's instructions. All assays were repeated at least three times.

### Tumor xenograft assay

1*10^6^ SW1990 cells were resuspended in 100ul culture medium and injected subcutaneously into each 4-week-old male nude mice. Mice were monitored daily for tumor growth and size and sacrificed after 2 weeks. All tumors were harvested, weighed, and then embedded in paraffin for the immunohistochemistry staining.

### Luciferase reporter assay

The 3′-UTR of GRB2 was amplified by PCR using cDNA from SW1990 cells and cloned into Hind III-MluI sites of the pMIR-REPORT miRNA expression reporter vector (Applied Biosystems). The mutant GRB2 3′-UTR was constructed to mutate three intermittent sites complementary to the miR-329 seed-region. The miR-329 mimics or control and luciferase reporter vector were co-transfected into SW1990 cells. 48h after transfection luciferase activities were measured with the dual-luciferase assay kit (Promega) according to manufacturer's instructions.

### Target genes analysis

We utilized the TargetScan as the tool for predicting the target genes of miR-329. GO analysis was performed to facilitate elucidating the biological implications of the target gene of miR-329. We downloaded the GO annotations from NCBI (http://www.ncbi.nlm.nih.gov/), UniProt (http://www.uniprot.org/) and the Gene Ontology (http://www.geneontology.org/). Fisher's exact test was applied to identify the significant GO categories and FDR was used to correct the p-values. Pathway analysis was used to find out the significant pathway of the differential genes according to KEGG database. We turned to the Fisher's exact test to select the significant pathway, and the threshold of significance was defined by *P* value and FDR. The MicroRNA-Target-Network was built based on the genes which are associated with proliferation and apoptosis. In the MicroRNA-Target -Network, the circle represents genes and the “V” shape represents MicroRNA hsa-miR-329. At the right of the Network, the genes are annotated by Gene ontology (GO). At the left of the Network, the genes are associated with Pathway analysis.

### Statistical analyses

All statistical analyses were performed using SPSS 16.0 software. The Student *t* test or one-way ANOVA was used when appropriate. The Pearson χ^2^ test was applied to analyze the correlation between miR-329 and clinicopathologic characteristics. Kaplan-Meier survival curves were compared using the log-rank test. Pearson bivariate correlation analysis was performed to test the association between miR-329 and GRB2 expression in tissues. *P* < 0.05 was considered statistically significant.

## SUPPLEMENTARY MATERIAL FIGURES AND TABLES








