# Supplementary material for: mir-329 restricts tumor growth by targeting grb2 in pancreatic cancer
Source: Oncotarget. 2016 Feb 14;7(16):21441–53. doi: 10.18632/oncotarget.7375 (PMC5008297; doi:10.18632/oncotarget.7375)
Supplement: Supplementary file 1 [file oncotarget-07-21441-s001.pdf]

mir-329 restricts tumor growth by targeting grb2 in pancreatic cancer

Supplementary Material

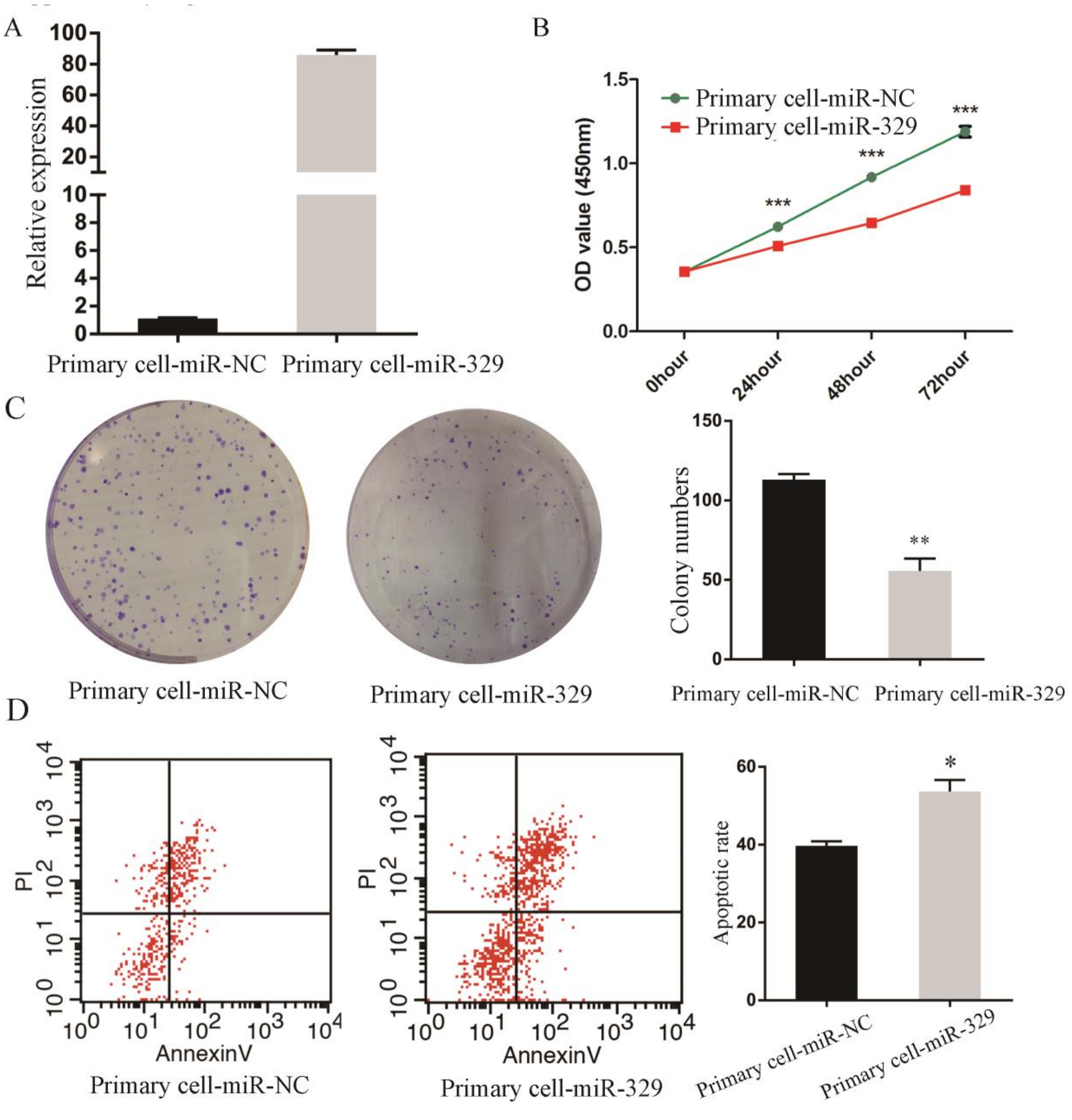

Supplementary Figure 1: MiR-329 inhibited proliferation and induced apoptosis of primary pancreatic cancer cells. A. MiR-329 expressions in primary pancreatic cancer cells were quantified by qPCR. B. Cell proliferation assay in primary pancreatic cancer cells

transfected with miR-329 mimics was shown. C. Colony formation assay and statistical analysis of primary pancreatic cancer cells transfected with miR-329 mimics were exhibited. D. Flow cytometry and apoptotic rate analyses of primary pancreatic cancer cells was shown. Standard deviation of the mean was plotted for bar charts. (* $P < 0.05$, ** $P < 0.01$, *** $P < 0.001$)

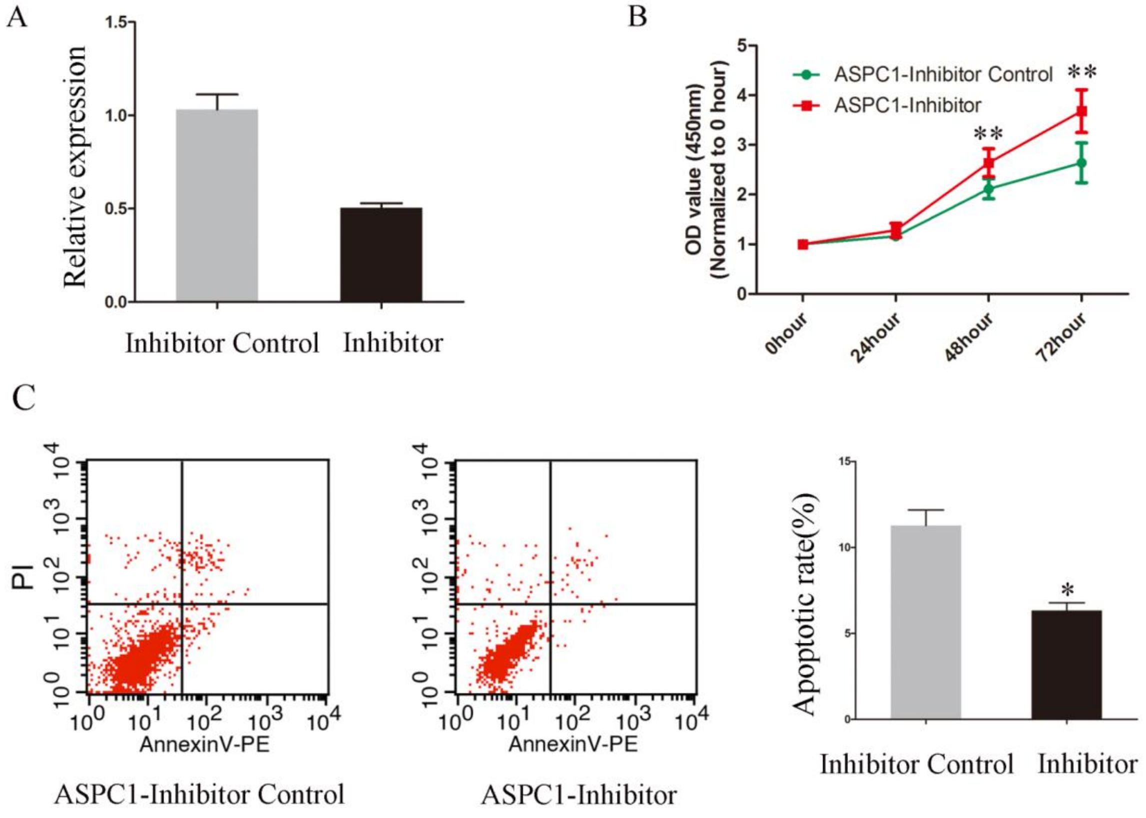

Supplementary Figure 2: miR-329 inhibitor promoted proliferation and decreased apoptosis in pancreatic cancer cell. A. miR-329 expression in cell line ASPC1 treated with its inhibitor or control was quantified by qPCR. B. Cell proliferation assay in ASPC1 transfected with miR-329 inhibitor were shown. C. The apoptotic response of ASPC1 cells treated with miR-329 inhibitor were shown. Standard deviation of the mean was plotted for bar charts. (* $P <$ 0.05, ** $P < 0.01$)
